# Genetic variations in *APPL2 *are associated with overweight and obesity in a Chinese population with normal glucose tolerance

**DOI:** 10.1186/1471-2350-13-22

**Published:** 2012-03-30

**Authors:** Shan Jiang, Qichen Fang, Weihui Yu, Rong Zhang, Cheng Hu, Kun Dong, Yuqian Bao, Chen Wang, Kunsan Xiang, Weiping Jia

**Affiliations:** 1 Shanghai Diabetes Institute, Shanghai Key Laboratory of Diabetes Mellitus, Shanghai Clinical Center for Diabetes, Department of Endocrinology and Metabolism, Shanghai Jiao Tong University Affiliated Sixth People's Hospital, Shanghai, China; 2 Department of Endocrinology & Metabolism, Shanghai Jiao Tong University Affiliated Sixth People's Hospital, 600 Yishan Road, Shanghai 200233, China

## Abstract

**Background:**

APPL1 and APPL2 are two adaptor proteins, which can mediate adiponectin signaling via binding to N terminus of adiponectin receptors in muscle cells. Genes encoding adiponectin and adiponectin receptors contribute to insulin resistance and the risk of obesity, and genetic variants of *APPL1 *are associated with body fat distribution. However, the association between genetic variations of *APPL2 *and metabolic traits remains unknown. In the current study, we aimed to test the impacts of *APPL2 *genetic variants on obesity in a Chinese population with normal glucose tolerance.

**Methods:**

We genotyped six single nucleotide polymorphisms (SNPs) in *APPL2 *in 1,808 non-diabetic subjects. Overweight and obesity were defined by body mass index (BMI). Obesity-related anthropometric parameters were measured, including height, weight, waist circumference, hip circumference. BMI and waist-hip ratio (WHR) were calculated.

**Results:**

We found significant evidence of association with overweight/obesity for rs2272495 and rs1107756. rs2272495 C allele and rs1107756 T allele both conferred a higher risk of being overweight and obese (OR 1.218, 95% CI 1.047-1.416, *p *= 0.011 for rs2272495; OR 1.166, 95% CI 1.014-1.341, *p *= 0.031 for rs1107756). After adjusting multiple comparisons, only the effect of rs2272495 on overweight/obesity remained to be significant (empirical *p *= 0.043). Moreover, we investigated the effects of these SNPs on obesity-related quantitative traits in all participants. rs2272495 was associated with BMI (*p *= 0.015), waist circumference (*p *= 0.006), hip circumference (*p *= 0.025) as well as WHR (*p *= 0.047) under a recessive model. Similar associations were found for rs1107756 except for WHR.

**Conclusion:**

This study suggests that genetic variations in *APPL2 *are associated with overweight and obesity in Chinese population with normal glucose tolerance.

## Background

In recent years, the worldwide prevalence of obesity has increased dramatically and has became a major global epidemic impacting on morbidity and mortality. According to data from World Health Organization (WHO), more than 1 billion adults are overweight worldwide and at least 300 million are clinically obese [1]. Although the developments of obesity are mainly attributed to environmental and behavioural factors such as a sedentary habit and overly rich nutrition, multiple genetic components do predispose to this disease, which is suggested by genome-wide association studies (GWAS) and genetic epidemiologic researches [2]. Up to now, GWAS have identified approximately 50 loci associated with obesity [3].

Adaptor protein, phosphotyrosine interaction, PH domain and leucine zipper containing 1 (APPL1) and Adaptor protein, phosphotyrosine interaction, PH domain and leucine zipper containing 2 (APPL2) are two adaptor proteins, which can mediate adiponectin signaling via binding to N terminus of adiponectin receptors in muscle cells [4-6]. They are both highly expressed in insulin-target tissues, including skeletal muscle, liver and adipose tissue [4,7]. APPL1 and APPL2 were found to share 54% identity in protein sequences and be encoded by gene on human chromosomes 3 and 12, respectively [6]. Our previous study has demonstrated that genetic variants of *APPL1 *are correlated with body fat distribution in Chinese type 2 diabetic patients [8]. However, to date, there is no study available concerning the association between genetic variations of *APPL2 *and metabolic traits. Therefore, we investigated the association of *APPL2 *with obesity and obesity-related quantitative traits in a Chinese non-diabetic population.

## Methods

### Participants

The present study included 1,806 non-diabetic adult individuals (age ≥ 19) of Han Chinese ancestry who participated in the community-based Shanghai Diabetes Studies [9]. The detailed inclusion and exclusion criteria for all subjects have been described previously [9]. Briefly, all the individuals were with normal glucose regulation, over 40 years old and with no family history of diabetes. And the normal glucose tolerance defined as fasting plasma glucose level < 6.1 mmol/l and 2 h plasma glucose level of 75 g OGTT < 7.8 mmol/l. BMI was used to assess generalized obesity according to the Chinese criteria that classified all individuals into three groups: normal weight (BMI < 24 kg/m^2^), overweight (24 ≤ BMI < 28 kg/m^2^) and obese (BMI ≥ 28 kg/m^2^) [10]. The clinical characteristics of three groups were given in Table 1. All study participants gave written informed consent, and study protocols were approved by the institutional review board of Shanghai Jiao Tong University Affiliated Sixth People's Hospital.

**Table 1:** Clinical characteristics of study population

	Normal Weight	Overweight	Obesity
Samples (n)	1046	601	159

Male/Female (n)	443/603	244/357	60/99

Age (years)	57.04 ± 12.76	57.49 ± 11.81	58.50 ± 11.37

BMI (kg/m^2^)	21.72 (20.27, 22.81)	25.61 (24.79, 26.64)	29.29 (28.48, 31.04)

Waist circumference (cm)	73.50 (69.00, 78.00)	84.00 (79.00, 89.00)	93.50 (88.00, 98.00)

Hip circumference (cm)	89.00 (85.00, 92.00)	95.50 (93.00, 99.00)	102.00 (99.00, 105.00)

WHR	0.83 (0.79, 0.87)	0.88 (0.84, 0.92)	0.91 (0.87, 0.92)

Abbreviation: *BMI *body mass index; *WHR *waist to hip ratio.

Data are shown as mean ± SD or median (interquartile range).

### Clinical measurement

All participants underwent detailed clinical investigations as described previously [9]. In brief, anthropometric parameters, including height, weight, waist and hip circumference were measured in all subjects. BMI was calculated as weight in kilograms divided by the square of height in meters and waist-to-hip ratio (WHR) was calculated as waist circumference (cm)/hip circumference (cm).

### Single nucleotide polymorphism (SNP) selection and genotyping

In the present study, we selected six tagging SNPs in *APPL2 *from the HapMap Phase 3 Chinese population including rs935251, rs10861360, rs1196744, rs1196768, rs3751191 and rs1107756 together with one non-synonymous SNP (rs2272495) in exon15. The figure S1 (see Additional file 1) shows linkage disequilibrium plot of all variants within *APPL2 *using CHB data of HapMap version 3 Release 27 and highlights the SNPs selected in our study. All the seven SNPs were genotyped by primer extension of multiplex products with detection by matrix-assisted laser desorption/ionisation time-of-flight mass spectroscopy using a MassARRAY platform (MassARRAY Compact Analyzer; Sequenom, San Diego, CA, USA). rs935251 is excluded because of genotyping failure and rs1196744 because of departure from Hardy-Weinberg equilibrium (*p *= 6.76E-56). The remaining five SNPs could tag 93% SNPs with a minor allele frequency (MAF) of > 0.1 from 18.9 kb upstream to 7.5 kb downstream of *APPL2 *based on an r^2 ^of ≥ 0.7. The call rates for rs2272495, rs10861360, rs1196768, rs3751191 and rs1107756 were 97.0%, 95.6%, 96.3%, 96.5% and 96.8%, respectively. The concordance rates based on 100 duplicates were over 99% for all these SNPs.

### In silico prediction of the functionality of APPL2 SNPs

PolyPhen2 (Polymorphism Phenotyping v2), a revised version of PolyPhen, is a tool which can predict the possible impact of missense mutations on the structure and function of encoded proteins. The prediction is based on performing various sequence and structure analyses. We assessed the functionality of coding variant (rs2272495) using PolyPhen2. The outputs of predictions are cataloged as benign, possibly damaging or probably damaging. PolyPhen2 is available at http://genetics.bwh.harvard.edu/pph2.

### Statistical analysis

*χ*^2 ^test was performed to estimate Hardy-Weinberg equilibrium for each variant before association analyses. Pairwise linkage disequilibrium was estimated for all DNA samples via calculating |D'| and r^2 ^using Haploview (version 4.2) [11]. Haplotype block structure was assessed by confidence interval algorithm [12]. Expectation-Maximization algorithm were used to estimated Haplotype frequencies by Haploview (version 4.2) [13]. The allelic and haplotype frequencies were compared using *χ*^2 ^tests between overweight and obese subjects and those with normal weight. Odds ratios (ORs) with 95% confidence intervals (CIs) were presented. Associations of SNPs with obesity-related measurements were assessed under a recessive model. Non parametric approach of rank transformation was performed to assess the contrasts results among three genotypes. Skewly distributed quantitative traits, including BMI, waist circumference, hip circumference and WHR were log_10 _transformed to approximate univariate normality before analysis. For adjusting multiple comparison, 10,000 permutations were performed to assess empirical *p *values using PLINK (version1.07) [14]. All statistical analyses were performed using SAS for Windows (version 8.0; SAS Institute, Cary, NC, USA) unless specified otherwise. A two-tailed *p *value < 0.05 was considered significant. We estimated study power using QUANTO. Assuming an additive model with the minor allele frequencies of 0.1, 0.2, 0.3 and 0.4 in our Chinese population, our sample size has 0.53, 0.77, 0.87 and 0.91 power to detect an OR of 1.25 at an α level of 0.05.

## Results

**Figure 1:**
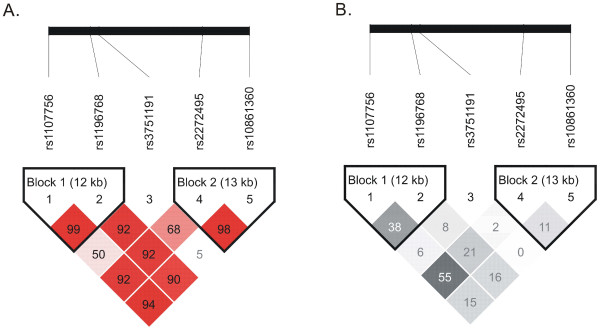
**Linkage disequilibrium maps for SNPs genotyped in *APPL2 *in the Chinese subjects with normal glucose tolerance**. A Shades of red demonstrate the strength of the pairwise linkage disequilibrium based on |D'| and numbers indicate the value of |D'| expressed as a percentage. B Shades of grey demonstrate the strength of pairwise linkage disequilibrium based on r^2^, and numbers indicate the value of r^2 ^expressed as a percentage.

Linkage disequilibrium analysis revealed that these five SNPs were in modest linkage disequilibrium and formed two haplotype blocks in this region (Figure 1).

We first examined the associations of these SNPs with the risk of overweight/obesity. Significant evidence of associations with overweight/obesity were observed for rs2272495 and rs1107756. rs2272495 C allele and rs1107756 T allele both conferred a higher risk of being overweight and obese (OR 1.218, 95% CI 1.047-1.416, *p *= 0.011 for rs2272495; OR 1.166, 95% CI 1.014-1.341, *p *= 0.031 for rs1107756). However, after adjusting multiple comparisons, only the effect of rs2272495 on overweight and obese remained to be significant (empirical *p *= 0.043). We also analysed the correlation between these SNPs and overweight/obesity after adjusting for age and sex. All the SNPs showed similar effects with or without adjustment (Table 2). In the haplotype association analyses, we compared the haplotype distributions between overweight/obesity and normal weight. We observed that the haplotype CG in block 1 (rs1107756-rs1196768) and the haplotype TC in block 2 (rs2272495-rs10861360) were associated with overweight/obesity (*p *= 0.041 and *p *= 0.018, respectively) (Table 3).

**Table 2:** Associations of *APPL2 *SNPs with overweight and obesity

SNP	Mayor/minor allele	Risk allele	Overweight + Obesity		Normal Weight		OR(95%CI)	*p *value	Empirical *p *value	OR(95%CI) #	*p *value #	Empirical *p *value #
									
			Risk allele frequencies	Genotype count 11/12/22^a^	Risk allele frequencies	Genotype count 11/12/22^a^						
rs1107756	T/C	T	0.652	313/326/91	0.616	391/473/154	1.166(1.014-1.341)	**0.031**	0.128	1.160 (1.009-1.333)	**0.037**	0.143

rs1196768	G/A	A	0.413	253/354/126	0.387	377/480/149	1.118(0.974-1.282)	0.112	0.375	1.118 (0.974-1.283)	0.113	0.376

rs3751191	C/T	T	0.135	551/169/15	0.126	767/228/13	1.086(0.890-1.325)	0.416	0.868	1.088 (0.891-1.329)	0.409	0.868

rs2272495	C/T	C	0.746	408/290/43	0.707	507/416/88	1.218(1.047-1.416)	**0.011**	**0.043**	1.216 (1.044-1.415)	**0.012**	0.052

rs10861360	C/T	T	0.245	415/266/45	0.227	604/339/58	1.104(0.942-1.294)	0.220	0.611	1.099 (0.939-1.285)	0.241	0.651

^a^11, major allele homozygotes; 12, heterozygotes; 22, minor allele homozygotes.

# adjust for age and sex.

Significant values are indicated in boldface.

**Table 3:** Associations of haplotypes consisting of two SNPs in the *APPL2 *region with overweight and obesity

Haplotype	Haplotype frequencies		*p *value
		
	Overweight + Obesity	Normal Weight	
Block 1 (rs1107756 - rs1196768)			

TA	0.413	0.386	0.105

CG	0.352	0.386	**0.041**

TG	0.235	0.228	0.647

Block 2 (rs2272495 - rs10861360)			

CC	0.499	0.480	0.289

TC	0.256	0.292	**0.018**

CT	0.244	0.226	0.219

Significant values are indicated in boldface.

We further investigated the effects of these SNPs at *APPL2 *on obesity-related measures, including BMI, waist circumference, hip circumference and WHR in all participants. We found that rs2272495 was associated with BMI (*p *= 0.015), waist circumference (*p *= 0.006), hip circumference (*p *= 0.025) as well as WHR (*p *= 0.047) under a recessive model (Table 4). Similar associations were found for rs1107756 except for WHR (see Additional file 2). The non parametric approach also showed the similar effect of rs2272495 on the BMI, waist circumference and hip circumference (*p *= 0.026, *p *= 0.015 and *p *= 0.033, respectively) (see Additional file 3). And rs1107756 was only associated with waist circumference (*p *= 0.033) using non parametric approach (see Additional file 4).

**Table 4:** Association of rs2272495 in *APPL2 *with obesity-related measures in all participants

rs2272495	TT(n = 131)	CT(n = 706)	CC(n = 915)	*p*
	
	Median (interquartile range)	Median (interquartile range)	Median (interquartile range)	TT + CT VS. CC
BMI (kg/m2)	22.806 (21.197, 24.844)	23.166 (21.193, 25.398)	23.459 (21.502, 25.932)	**0.015**

waist circumference (cm)	77.000 (71.000, 83.000)	78.000 (71.000, 84.000)	79.000 (72.000, 86.000)	**0.006**

hip circumference (cm)	91.000 (87.000, 95.000)	91.500 (87.000, 96.000)	92.000 (88.000, 97.000)	**0.025**

WHR	0.849 (0.800, 0.898)	0.849 (0.809, 0.895)	0.857 (0.809, 0.906)	**0.047**

Abbreviation: *BMI *body mass index; *WHR *waist to hip ratio.

*P *values were assessed under a recessive model.

Obesity related measures under investigation were log transformed before analysis.

Significant values are indicated in boldface.

## Discussion

In the present study, we tested five SNPs in *APPL2 *and identified two novel risk-conferring SNPs, rs2272495 and rs1107756, for overweight and obesity in non-diabetic individuals. We also found these SNPs, especially rs2272495, were associated with obesity-related measures in these subjects.

APPL2 is an isoform of APPL1 and can form a dimer with APPL1 [15,16]. APPL1 is a critical regulator in both adiponectin and insulin signaling pathway. APPL1, which binds directly to adiponectin receptors (AdipoR1 and AdipoR2), can positively mediate adiponectin signaling and phosphorylate its downstream molecules, leading to increased glucose uptake and fatty acid oxidation in muscle cells. In addition, APPL1 can enhance the synergistic effect of adiponectin on insulin-stimulated Akt phosphorylation and mediate the cross-talk between adiponectin and insulin signaling pathways [5]. Recently, APPL2 has also been suggested to play an important role in adiponectin and insulin signaling as well as the cross-talk between these two pathways. APPL2 can negatively regulate adiponectin signaling by competing with APPL1 in binding to AdipoR1 in muscle cells. Suppression of APPL2 can promote adiponectin-stimulated glucose uptake and fatty acid oxidation [4]. Although, unlike APPL1, APPL2 does not directly interact with the catalytic subunit of PI3-kinase and Akt2 which are key kinases in the PI3-kinase pathway downstream of the insulin receptor [15,16], APPL2 can suppress insulin signaling by inhibiting the interaction between APPL1 and the components of insulin signaling. Thus, suppression of APPL2 greatly enhanced the sensitizer effect of adiponectin on insulin [4].

Previously, a link between genes involved in adiponectin signaling and metabolic traits has been suggested by several studies. The adiponectin gene contributes to variances in the plasma adiponectin levels and insulin resistance index and predisposes to obesity and type 2 diabetes [17-19]; the genes encoding adiponectin receptors are associated with insulin resistance, liver fat and the risk of type 2 diabetes [20,21]; SNPs in *APPL1 *are found to be correlated with body fat distribution in Chinese patients with type 2 diabetes [8]. Taken together all these findings, it is conceivable that the gene encoding APPL2, which also participates in adiponectin signaling and indirectly mediates insulin signaling, may also exert an effect on metabolic traits. Consistent with this presumption, our study found an association of *APPL2 *with overweight/obesity in non-diabetic individuals of Han Chinese ancestry, with rs2272495 C allele and rs1107756 T allele conferring a higher risk of being overweight and obese. It should be noted that obesity is a complex disease that affected by multiple genes and environmental factors. The effect of one genetic variant on obesity and obesity-related traits was limited. Therefore, it is convincible that rs2272495 may affect obesity-related traits according to our results in the Table 4. It is tempting to propose that rs2272495 might be a causal variant for overweight/obesity since it is a strong signal detected in our study and a non-synonymous variant which substitutes valine to alanine. Although this variant is predicted to be benign by PolyPhen2, whether it has effect on protein function in vivo remains unknown and should be further elucidated by mechanic studies. The rs2272495 and rs1107756 were in same haplotype block and displayed D' = 0.92 and r^2 ^= 0.55 in our study population. Based on 0.55 r^2^, it is a relatively strong LD. Thus, the associations observed for the rs1107756 may be a marker to reflect the association signal for rs2272495. Nevertheless, the possibility that other SNPs may be etiological cannot be excluded. The mechanisms underlying the association between *APPL2 *and obesity are still unclear, and need to be further illuminated by functional studies.

Some limitations should be noted in the present study. Firstly, we tested the same association of the SNP with adiposity multiple times without correcting for these additional tests in the Table 4. In fact, after Bonferroni correction, rs2272495 was still associated with waist circumference (*p *= 0.024). Secondly, regarding the Table 4, rs2272495 were associated with obesity-related measures. However, when stratifying according to the 3 subgroups (normal weighted, overweight and obese), no significant association was observed in subgroups (see Additional file 5). As the population included in the present study aged all above 40 years old and age factor may influence the prevalence of obesity, the rate of overweight/obese in our manuscript was higher than reports on obesity in China. Actually, the obesity related phenotypes such as BMI presented in our study were skewed distributed. According to these results (Table 4 and see Additional file 5), we cannot draw solid conclusions that rs2272495 were associated with obesity-related measures in our subjects. The associations between rs2272495 and obesity related phenotypes may only reflect the association between rs2272495 and overweight/obesity. Thirdly, although we found associations of rs2272495 and rs1107756 with overweight/obesity and obesity-related measurements, we had not the opportunity to perform a replication another independent sample. Further replications in other cohorts are needed to confirm the influence of genetic variation within this locus on overweight/obesity or metabolic traits in the Chinese population.

## Conclusion

Our data suggest that genetic variations in *APPL2 *are associated with overweight and obesity in the Chinese subjects with normal glucose tolerance. Further investigations are needed to confirm our findings and demonstrate the mechanisms underlying such association.

## Abbreviations

SNP: Single nucleotide polymorphisms; BMI: Body mass index; WHR: Waist-hip ratio; WHO: World Health Organization; GWAS: Genome-wide association studies; APPL1: Adaptor protein, Phosphotyrosine interaction, PH domain and leucine zipper containing 1; APPL2: Adaptor protein, Phosphotyrosine interaction, PH domain and leucine zipper containing 2; OGTT: Oral glucose tolerance test; MAF: Minor allele frequency; OR: Odds ratios; CI: Confidence interval; AdipoR1: Adiponectin receptor 1; AdipoR2: Adiponectin receptor 2.

## Competing interests

The authors declare that they have no competing interests.

## Authors' contributions

SJ and QF participated in genotyping, performed statistical analysis and drafted the manuscript. WY participated in genotyping and revised the manuscript. RZ prepared the DNA samples and participated in genotyping. CH designed the study and revised the manuscript. KD participated in sample collection and prepared the DNA samples. CW participated in the clinical study and revised the manuscript. YB participated in clinical study and contributed to discussion. KX contributed to discussion. WJ supervised the study and revised the manuscript. All authors read and approved the final manuscript.

## Pre-publication history

The pre-publication history for this paper can be accessed here:

http://www.biomedcentral.com/1471-2350/13/22/prepub

## Supplementary Material

Additional file 1 **Linkage disequilibrium plot of SNPs within *APPL2 *using CHB data of HapMap version 3 Release 27**. This PDF file contains |D'| measures of linkage disequilibrium for each SNP pair within *APPL2 *using CHB data of HapMap version 3 Release 27. Click here for file

Additional file 2 **Association of rs1107756 in *APPL2 *with obesity-related measures in all participants**. This Microsoft Excel file contains detailed information of association between rs1107756 in *APPL2 *and obesity-related measures, including BMI, waist circumference, hip circumference and WHR, under a recessive model. Click here for file

Additional file 3 **Effect of rs2272495 on obesity-related measures in all participants**. This Microsoft Excel file contains detailed information of the differences among three genotypes of rs2272495 for every obesity-related measures using non parametric approach of rank transformation. Click here for file

Additional file 4 **Effect of rs1107756 on obesity-related measures in all participants**. This Microsoft Excel file contains detailed information of the differences among three genotypes of rs1107756 for every obesity-related measures using non parametric approach of rank transformation. Click here for file

Additional file 5 **Association of rs2272495 in *APPL2 *with obesity-related measures in 3 subgroups (normal weighted, overweight and obese)**. This Microsoft Excel file contains detailed information of association between rs2272495 in *APPL2 *and obesity-related measures, in separate groups (normal weighted, overweight and obese), under a recessive model. Click here for file
