# Supplementary material for: Genetic variations in APPL2 are associated with overweight and obesity in a Chinese population with normal glucose tolerance
Source: BMC Med Genet. 2012 Mar 30;13:22. doi: 10.1186/1471-2350-13-22 (PMC3368742; doi:10.1186/1471-2350-13-22)
Supplement: Additional file 1 — Linkage disequilibrium plot of SNPs within APPL2 using CHB data of HapMap version 3 Release 27. This PDF file contains |D'| measures of linkage disequilibrium for each SNP pair within APPL2 using CHB data of HapMap version 3 Release 27. [file 1471-2350-13-22-S1.PDF]

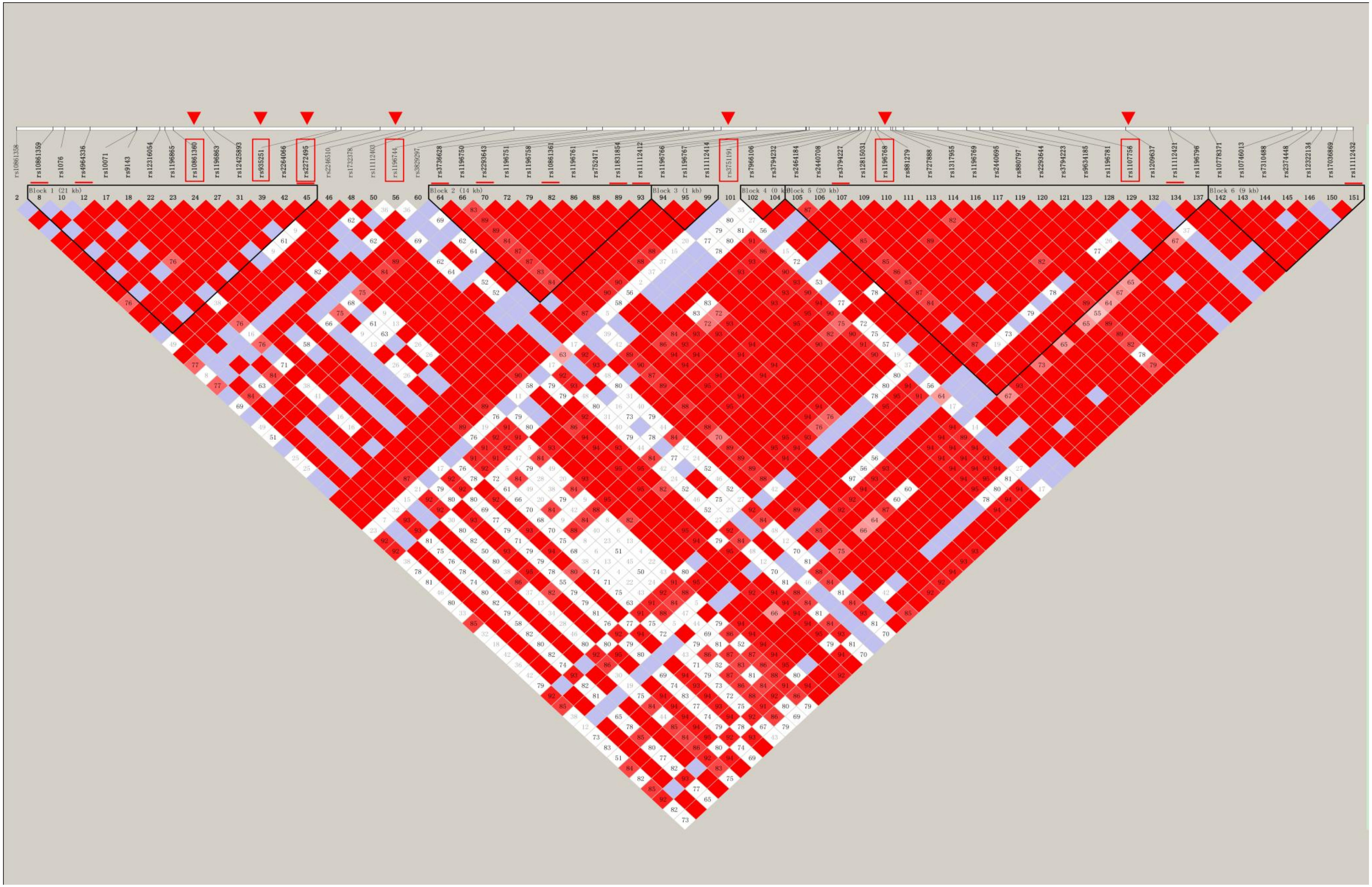

Figure S1 Linkage disequilibrium plot of SNPs within *APPL2* using CHB data of HapMap version 3 Release 27. Shades of red indicate the strength of pairwise linkage disequilibrium based on |D'|. Numbers within the diamonds are |D'| of each SNP pair. Selected six tagging SNPs together with one non-synonymous SNP in our study are marked with the box. And the SNPs tagged by rs2272495 were underlined in the figure.
